# Musculoskeletal Pain during the Menopausal Transition: A Systematic Review and Meta-Analysis

**DOI:** 10.1155/2020/8842110

**Published:** 2020-11-25

**Authors:** Chang-bo Lu, Peng-fei Liu, Yong-sheng Zhou, Fan-cheng Meng, Tian-yun Qiao, Xiao-jiang Yang, Xu-yang Li, Qian Xue, Hui Xu, Ya Liu, Yong Han, Yang Zhang

**Affiliations:** ^1^Departments of Orthopaedic Surgery, Xijing Hospital, Fourth Military Medical University, Xi'an, Shanxi 710032, China; ^2^Departments of Urinary Surgery, Xijing Hospital, Fourth Military Medical University, Xi'an, Shanxi 710032, China; ^3^Department of Thoracic Surgery, Tangdu Hospital, Fourth Military Medical University, Xi'an, Shanxi 710038, China; ^4^Department of Neurobiology and Collaborative Innovation Center for Brain Science, School of Basic Medicine, Fourth Military Medical University, Xi'an, Shanxi 710032, China; ^5^Department of Out-Patient, Xijing Hospital, Fourth Military Medical University, Xi'an, Shaanxi 710032, China

## Abstract

Musculoskeletal pain (MSP) is one of the most severe complaints in women undergoing menopause. The prevalence of MSP varied when taking the menopausal state and age factor into consideration. This study investigated the prevalence of MSP in perimenopausal women and its association with menopausal state. The MEDLINE, Embase, Web of Science, and PubMed databases were searched from inception to July 2020, and 16 studies were retrieved for the current meta-analysis. The primary outcome measure was the MSP Odds Ratio (OR). The estimated overall prevalence of MSP among perimenopausal women was 71% (4144 out of 5836, 95% confidence interval (CI): 64%-78%). Perimenopausal women demonstrated a higher risk for MSP than premenopausal ones (OR: 1.63, 95% CI: 1.35-1.96, *P* = 0.008, *I*^2^ = 59.7%), but similar to that in postmenopausal ones (OR: 1.07, 95% CI: 0.95–1.20, *P* = 0.316, *I*^2^ = 13.4%). The postmenopausal women were at a higher risk of moderate/severe MSP than the premenopausal ones (OR: 1.45, 95% CI: 1.21-1.75, *P* = 0.302, *I*^2^ = 16.5%) or the perimenopausal ones (OR: 1.40, 95% CI: 1.09–1.79, *P* = 0.106, *I*^2^ = 55.4%). In conclusion, the perimenopause is a state during which women are particularly predisposed to develop MSP. As to moderate to severe degrees of MSP, the odds increase linearly with age, from premenopause to peri- and then to postmenopause.

## 1. Introduction

Musculoskeletal pain (MSP) poses a heavy burden on both medical expenses and patients' qualities of lives and affects over half of the female population at midage [1–4]. Generally, MSP is a predominant manifestation of musculoskeletal disorders (MSDs) and involves soft tissues including muscles, tendons, and nerves [5]. However, such an association is not necessarily true when it comes to perimenopausal women. In clinical settings, menopausal states are defined according to the State of Reproductive Aging Workshop (ATRAW) criteria, which are based on the menstrual history of the women. The premenopausal state is defined as with regular cycles of stable cycle length (21-35 days). A perimenopausal woman is confirmed if her menstrual cycles have been irregular for over one year or her last menstrual period occurred at least 2 but less than or equal to 11 months amenorrhea. Women who had not menstruated for at least 12 months are considered at the postmenopausal state [6]. Previously, MSP was listed as one of the predominant menopausal symptoms, together with others, including hot flashes, mood changes, and depression [4, 7, 8].

Although MSP is one of the salient symptoms that occur during menopausal phases [9], its prevalence varies among midlife women at different menopausal states, especially in the transition between the perimenopausal and postmenopausal states. In some studies, it was reported that the prevalence of musculoskeletal pain reached the peak at the early postmenopause state [3], while MSP prevalence was reported to increase along the perimenopause phase [10]. While in another study encompassing large middle-aged women, a significant salience in the MSP prevalence was observed especially in peri- and postmenopausal women, and the MSP was significantly associated with menopausal state [11].

Although menopausal women complaining of MSP took up a large proportion of outpatient visits, yet, the majority of them turned out to have no significant imaging findings in magnetic resonance imaging (MRI). They often did not require specific medical interventions. As is illustrated in one study investigating the sex-based differences in the consequences of MSP, menopausal women in the queue frequently reported the necessity of healthcare due to MSP, but rarely involving work- or disability-leave [12]. Meanwhile, a direct relationship was proposed between the menopause state and the MSP intensity [13]. When pain intensity was differentiated as mild, moderate, and severe degrees, MSP prevalence was showed a different pattern among three menopausal states.

Despite all these reported data, it remains ambiguous in terms of the relationship between the MSP prevalence and menopausal states. In addition to the menopausal states, both health state and health-related behavioral factors including smoking, Body Mass Index (BMI), and depression were associated with MSP [14–16].

Thus, we attempted to investigate whether the menopausal state is an independent factor for the MSP. We assigned premenopausal women as the control group to measure the basic MSP prevalence. Given the lack of longitudinal investigations on the MSP prevalence, we undertook a meta-analysis based mainly on the cross-sectional studies. The systematic review and meta-analysis were conducted to investigate (1) the overall MSP prevalence in perimenopausal women and (2) the association between the menopausal state and the MSP prevalence.

## 2. Methods

### 2.1. Search and Selection of Relevant Studies

We performed the literature search without language restriction using the MEDLINE (Ovid), Embase (Elsevier), Web of Science, and PubMed databases from the inception to July 2020. Databases were queried for studies conducted on the basis of menopausal symptoms and MSP. Menopausal states were classified as premenopausal, perimenopausal, or postmenopausal ones according to the ATRAW criteria. Combinations of search terms “musculoskeletal pain” and “muscle or joint pain” with “climacteric”, “menopause”, and “female hormones” have been used. Exclusion terms as “premature ovarian failure” were applied with the restriction “human” and “female”. Complete search algorithms for each database were available in the Database Search Algorithms supplement section.

Studies were included in the qualitative review if they met the following criteria: (1) those containing primary data of women sufficient for meta-analysis; (2) those using the STRAW criteria to define menopausal states [16]; (3) those reporting at least one estimated MSP prevalence for the whole course of menopause or MSP prevalence of any menopausal states; and (4) those passing critical appraisal. In addition, studies reporting on the prevalence of MSP were only included if the pain was assessed using a previously validated instrument or a de novo instrument designed by occupational medicine experts. We excluded studies without differentiating the menopausal state or with irrelevant PICO records. Articles were also excluded if they were reviews, editorials, or case reports. All retrieved articles were carefully read, and citations were screened for articles missed during the searching procedure. Besides, the principal investigators of ongoing trials were contacted about imminent publications.

### 2.2. Data Extraction and Quality Assessment

Data from each study reporting the estimates of prevalence were initially extracted. The extracted data contained the following items: study design, geographic location, sample size, mean age of the subjects, and instruments used for diagnosis or screening. Instruments used to evaluate diagnosis and pain were recorded as well. We extracted data (prevalence of menopausal symptoms at different menopausal states) for MSP-associated symptoms or complaints by the menopausal state, with the premenopausal state being the referent group in all cases. Pain symptoms were assessed by questionnaires including in the standardized measurement scales MRS and MENQOL. For studies using the de novo instruments, the diagnosis of MSP was made on the presence of chronic muscle or joint pain in the past 4 to 6 weeks. Data extracted from each study reported outcomes of ergonomics assessments or interventions, such as body mass index (BMI) and geographic location. The checklist of critical appraisal obtained from the 2014 Reviewers' Manual published by the Joanna Briggs Institute was used to assess the quality of each study that reported the estimation of prevalence (Table S1).

Publication bias was assessed using the approach proposed by Egger's group [17]. The Egger's approach was based on the funnel plot in which the standardized effect estimation was regressed on a measure of the precision (1/SE).

### 2.3. Data Analysis

The Meta-analysis of Observational Studies in Epidemiology (MOOSE) guidelines were followed in the current study. A software program (StataMP 14) [18, 19] was utilized for all statistical analyses. The primary outcome was calculated using the odds ratio (OR) of menopausal state for MSP. Considering the remarkable heterogeneity between studies, random effect models [20] were used to estimate the overall summarized (log) odds ratios and 95% confidence interval (CI) of the menopausal state on MSP symptoms.

Possible heterogeneity was assessed by using the *I*^2^ and was assessed for statistical significance using the *Q* statistic. A significant difference was considered at *P* < 0.05. The objective to study this topic was driven by questions from patients in the target population on their odds on MSP.

## 3. Results

**Figure 1:**
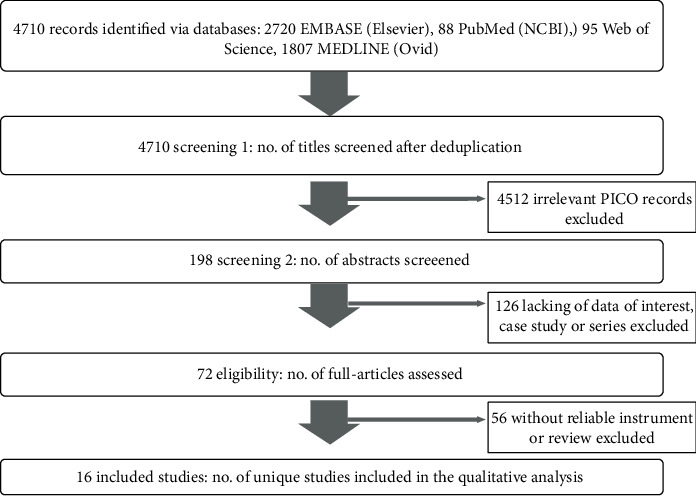
Preferred Reporting Items for Systematic Reviews and Meta-Analyses (PRISMA) Flow Diagram of the Systematic Literature Search and Review Protocol. NCBI: National Center for Biotechnology Information; PICO: population, injury, or context.

The search returned 4710 unique items. Most were discarded due to irrelevant records or involving MSP in the general population (*N* = 5836). Others were discarded due to a lack of primary data of interest (*n* = 126) or the lack of a valid and reliable instrument used for evaluation (*n* = 56). Sixteen studies met the inclusion criteria and were included in the current study, among them, 2 were longitudinal, and 14 were cross-sectional. Among the 14 cross-sectional studies, 12 reported the MSP prevalence among women at menopausal states (Figure 1). All the included studies were published between 1997 and 2020 in 12 countries (Table 1).

### 3.1. Estimation of the MSP Prevalence in Perimenopausal Women

**Figure 2:**
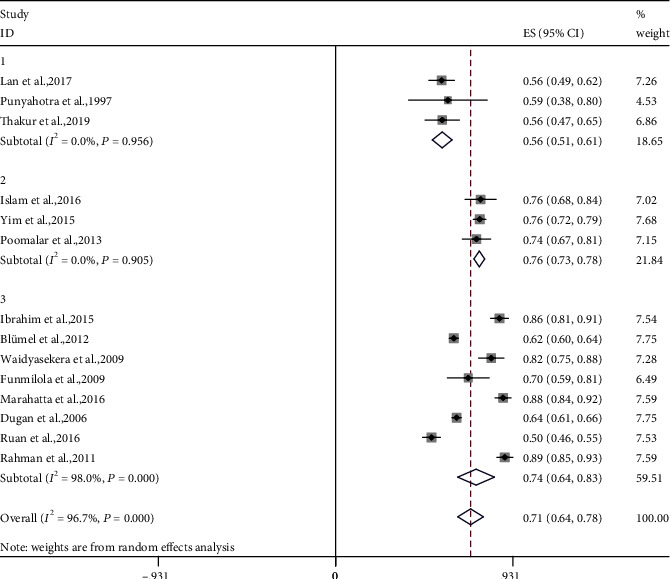
Meta-analyses of the estimated MSP prevalence in perimenopausal women. Fourteen studies were included in the analysis. ES (95% CI): effect sizes, (95% confidence interval).

MSP prevalence during perimenopause was reported in 14 cross-sectional studies. All 14 studies measured menopausal symptoms (including MSP) using validated questionnaires. The estimated overall MSP prevalence reached 71% (4144 of 5836) (95% confidence interval (CI): 64%-78%). Reported MSP prevalence ranged from 50% to 89%. The heterogeneity was considerable for all crude analyses (*I*^2^ = 96.7%, Figure 2). Consequently, subgroup analyses were performed by the measurement scales.

MSP-based questionnaire was used in four studies. De novo questionnaires were used to ask subjects if they had been distressed by chronic pain of the muscle or joint in the past 4 to 6 weeks. For these studies, the estimated MSP prevalence was 53% (95% CI: 49%-56%, *I*^2^ = 0.0%, *P* = 0.464).

The Menopause-specific Quality of Life Questionnaire (MENQOL) scale was used in 3 studies [21]. For these studies, each of the domains included physical, psychological, and sexual aspects. Women reported whether they had experienced each symptom listed in the scale including muscle and joint pain in the last 4 weeks. For these women, the estimated MSP prevalence was 80% (95% CI: 73%-78%, *I*^2^ = 0.0%, *P* = 0.905).

The Menopause Rating Scale (MRS) was used in 7 studies [22]. Women were asked whether they had experienced the 11 menopausal symptoms shown in the MRS, including muscle and joint discomfort, in the previous month (30 days). Based on MRS, the estimated MSP prevalence was 77% (95% CI: 67%-87%, *I*^2^ = 97.9%, *P* < 0.001).

After evaluating the synthesized MSP prevalence and heterogeneity in subgroup analysis, we found that the MSP prevalence was higher when measured using MRS, and lower with MENQOL or de novo instruments.

### 3.2. The MSP Prevalence among Different Menopausal States

Two longitudinal studies described the odds ratios of MSP during the perimenopause and the premenopause (total *N* = 842) [9, 15]. The odds on the presence of MSP during the perimenopause significantly increased when compared to the premenopause (OR = 1.6, 95%, CI = 1.22 − 1.992, *P* < 0.01). No between-study heterogeneity (*P* = 0.937) was found, and thus moderate analyses were not performed.

**Figure 3:**
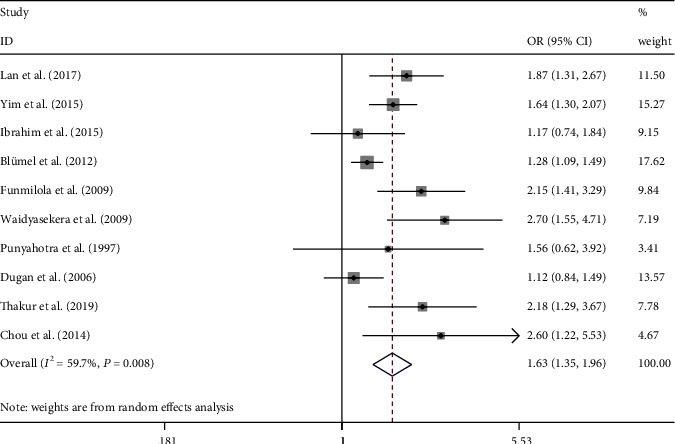
Meta-analysis of the estimated MSP prevalence between premenopausal and perimenopausal women. Ten studies were included in the analysis. OR (95% CI): odds ratio (95% confidence interval).

**Figure 4:**
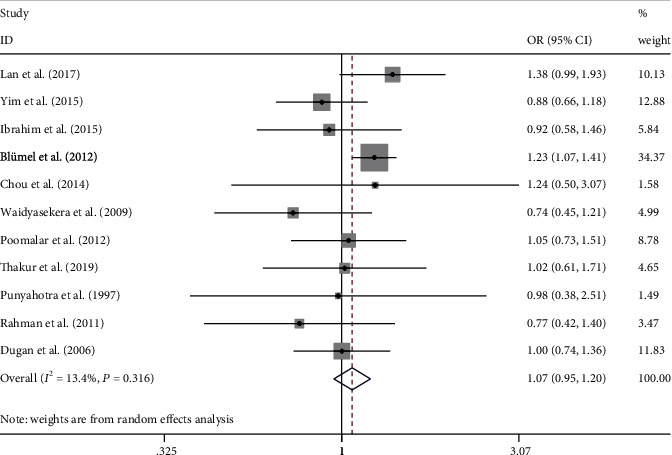
Meta-analysis of the prevalence estimates of MSP between perimenopausal and postmenopausal women. Eleven studies were included in the analysis. OR (95% CI): odds ratio (95% confidence interval).

The occurrence of MSP in the peri- and premenopausal states was compared in 10 studies. Compared with women in the premenopausal state, the odds on MSP in perimenopause women were significantly higher than in premenopausal women (Figure 3, OR = 1.63, 95% CI = 1.35 − 1.96, *P* = 0.008, *I*^2^ = 59.7%). Significant between-study heterogeneity among studies was detected. The heterogeneity was unrelated to the mean age of the participants or the questionnaire that was used. The occurrences of MSP in the perimenopause and postmenopausal women were compared in 13 studies. No significant difference in the odds of MSP was noted between the peri- and postmenopausal women (Figure 4, OR = 1.07, 95% CI = 0.95–1.20, *P* = 0.316, *I*^2^ = 13.4%). In this analysis, there was no significant between-study heterogeneity, and moderator analysis was not performed.

### 3.3. The Prevalence of Moderate/Severe MSP among Different Menopausal States

**Figure 5:**
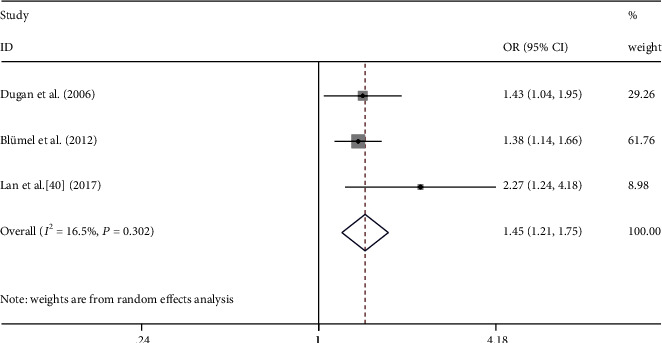
Meta-analysis of moderate/severe MSP between premenopausal and perimenopausal women. Three studies were included in the analysis. OR (95% CI): odds ratio (95% confidence interval).

The occurrences of moderate/severe MSP in the peri- and premenopausal states were compared in 3 studies. The odds of moderate/severe MSP in perimenopause women were significantly higher than those in the premenopausal women (Figure 5, OR = 1.45, 95% CI = 1.21 − 1.75, *I*^2^ = 16.5%). In this analysis, there was no significant between-study heterogeneity (*P* = 0.302), and moderator analysis was not performed.

**Figure 6:**
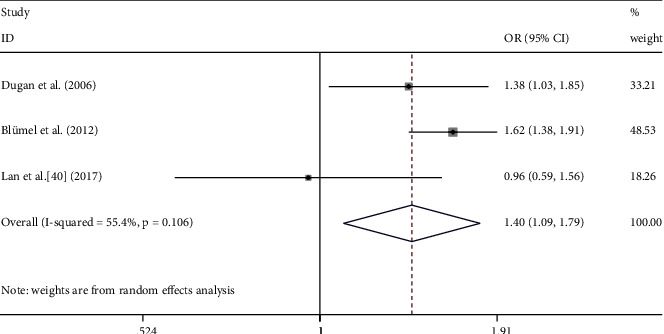
Meta-analysis of moderate/severe MSP between premenopausal and perimenopausal women. Three studies were included in the analysis. OR (95% CI): odds ratio (95% confidence interval).

The occurrences of moderate/severe MSP between the perimenopause and the postmenopausal were compared in three studies. A significant difference in the incidence moderate/severe MSP was found in the transition from the peri- to the postmenopausal states (Figure 6, OR: 1.40, 95% CI: 1.09–1.79, *I*^2^ = 55.4%). In this analysis, there was no significant between-study heterogeneity (*P* = 0.106), and moderator analysis was not performed.

**Figure 7:**
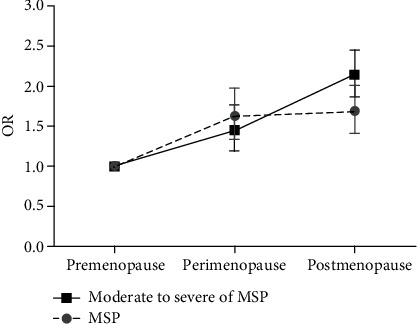
Calculated ORs for MSP degrees among different menopausal women. Premenopause OR is set as reference OR 1.00 group.

Synthesizing the calculated ORs for MSP of moderate to severe degree as compared to the premenopause as the reference OR group (1.00), we came out a graph on MSP (rated as moderate to severe degree) trends in different menopausal states (Figure 7). Across three menopausal states, the odds on MSP were increased significantly from pre- to perimenopausal state and then to postmenopausal state, deviating from the trend of MSP degrees at the perimenopausal state.

### 3.4. Influence of BMI and Other Emotional Factors on MSP

BMI was assessed in several studies included in the meta-analysis, most of which indicated that BMI was an independent risk factor for MSP. Lynnette's group performed univariate logistic regression analysis and found that women complaining of back pain, joint stiffness, and bone pain demonstrated a significantly higher BMI compared with that of women without pain complaints [23]. Similarly, Anny's group assessed pain intensity in menopausal women at different BMI levels using the visual analog scale (VAS). The results suggested that women whose BMI exceeded 30 kg/m2 were accompanied by significantly higher VAS scores than others [24]. Furthermore, it has been suggested that a high severity and frequency of aches and stiff joints were associated with higher mean values of BMI [9, 25]. In terms of confounding effects of BMI on the menopausal state, logistic regression models were used to assess both factors of BMI and the menopausal state to the presence of MSP during the menopause [14]. They found that although BMI might be related to pain symptoms in the early and late perimenopausal states, a significant difference still existed between postmenopausal and premenopausal women following considering BMI factors.

Other symptoms, such as sleep disruption, anxiety, and depressed mood during the menopausal transition, were also linked to pain symptoms. Females who experienced sleep disruption would increased pain severity [15]. Also, anxiety and depressed mood symptoms were associated with MSP symptoms [26]. One study revealed that negative moods towards age can explain a similar proportion of MSP in postmenopausal state [27]. However, these symptoms may be the results of pain experiences; in turn, they may also be underlying menopausal-related factors contributing to pain perception.

### 3.5. Publication Bias

Significant publication bias was revealed in the pre- vs. perimenopausal state analysis (intercept of the regression *a* = 2.5, *t* = 4.67, *P* = 0.037) but not the peri- vs. postmenopausal state analysis (intercept of the regression *a* = −1.98, *t* = 13.54, *P* = 0.079) (Figure S1-S2).

## 4. Discussion

This systematic review and meta-analysis on MSP during the menopausal age revealed (1) odds of MSP were salient among perimenopausal women; (2) the perimenopause women were particularly vulnerable to develop MSP compared to the premenopause; (3) the postmenopausal women were particularly vulnerable to develop moderate to severe MSP compared to the pre- and perimenopause women.

Our study on the basis of recent work demonstrated a high MSP prevalence among perimenopausal women. Previous studies have also demonstrated higher severity and frequency of aches in the musculoskeletal system were associated with negative mood [16]. Especially for the perimenopausal women, MSP has emerged as an enormous health burden, both physically and psychologically. To illustrate, we found the MSP prevalence among perimenopausal women reached 71%. In further meta-analysis on MSP across different menopausal states, we found an odds ratio of 1.63 on MSP in the perimenopause when compared to the premenopause. The odds on MSP were not increased significantly in the postmenopause compared with the perimenopausal phase as reference. These findings of increased odds of MSP in perimenopause, as measured by symptom clinical interviews, were consistent with many prior cross-sectional and studies [28]. When it comes to negative moods in MSP, as most studies included in our analysis were cross-sectional, we were not able to determine which came first, the pain or the depression. Further longitudinal studies would be investigated to compare the change in depression over time in association with pain factors with the menopause states.

Many studies included in our analysis indicated that factors like BMI and aging were also associated with MSP. The potential effects of BMI, aging, and pain intensity were related to outcome. While the etiology of BMI should be determined, its existence did not render the present systematic review and meta-analysis. It was shown that the association between BMI and MSP was weaker than that of the postmenopausal state [29]. In addition, the magnitudes of all study estimates were high enough to be meaningful, such that the overall effect estimate was informative, irrespective of variability. Aside from BMI, age was perhaps the most significant covariate. It was reported that the incidence of chronic low back pain increases with increasing age [30]. In one study, after adjusting for the age factor, those who were in perimenopausal state still reported significantly more aches and pains compared with those of premenopausal women [14].

Although the overall MSP prevalence showed no significant increase from peri-to postmenopausal state (OR = 0.95, 95% CI = 0.77 − 1.17), yet when differentiating the prevalence by pain degree, we found that odds on MSP of moderate to severe degree increased almost linearly with aging, from premenopausal state to perimenopausal state then to postmenopausal state. Such a trend is parallel with the prevalence of MSDs with aging [31], indicating that for menopausal women, moderate to severe degree of MSP may be of more diagnostic significance for the MSDs compared with MSP rated as any degree.

One concerning of the salient MSP in the menopause was a decline in bone health [32, 33]. Although slight changes in bone mineral density were observed during the premenopausal and early perimenopausal phases [34, 35], no consistent associations were found in the study exploring the relationships between sex hormonal and pain among middle-aged females [36]. Actually, the perimenopause was characterized by notable fluctuations in the levels of sex hormones, as opposed to a steady decrease [32, 37, 38]. Moreover, fluctuations in the levels of sex hormones have been shown to be associated with the central nervous system [39]. Consequently, we hypothesized that the synthesis of estrogen and fluctuation in blood levels during the perimenopausal phase might change the perception of pain in women and contribute to the salient occurrence of MSP among them. Nevertheless, further prospective studies of midlife women would be needed to better demonstrate the trajectory of MSP presence across all phases of the menopause and the hormonal influences on pain modulation.

It should be noted that heterogeneity was high for all analyses, especially for overall MSP prevalence when interpreting the results of the quantitative synthesis. The heterogeneity can be partly explained by differences in the sensitivity of the instruments used for MSP evaluation, occupation, and geographic location among study samples. Although differences in these variables were grossly evident, determining whether any one variable independently and significantly accounts for the heterogeneity was difficult because there was an insufficient number of studies for meta-regression.

## 5. Limitations

Despite the significant strengths of this systematic review and meta-analysis, this study contained several limitations. The first point was selection bias in which among 16 cross-sectional studies reporting the MSP prevalence in perimenopausal women, only 7 were analytical cross-sectional studies. The remaining included descriptive studies without reference groups. The second point was misclassification bias because primary data were collected through self-report methods. The third limitation was instrumental bias. MSP prevalence data in the study were subitems collected from instruments that mainly assessed menopausal symptoms.

## 6. Conclusions and Perspectives

In conclusion, this study came up with a possible potential association between the menopausal state and MSP. Specifically, MSP prevalence in perimenopausal women was remarkable. The odds on developing MSP increased from premenopause to perimenopause, but not from perimenopause to postmenopause. However, the odds on developing moderate to severe degree of MSP were increased linearly from premenopause to perimenopause and then to postmenopause. The implications of such trends are of great significance for future studies in recognition that perimenopausal state may represent an independent risk factor for MSP of mild degree. Realizing such a characteristic prevalence MSP across different menopausal states can also promote efficiency in identifying MSP among menopausal women in the orthopaedic outpatient clinics after taking into account their changing biology. Nevertheless, future studies are needed to provide convincing evidence of the associations between the menopausal state and MSP and to identify the specific risk factors for developing MSP in perimenopause. Moreover, more clinical evidence would be needed to determine if the long-term trajectories of mental burden resulted from pain in women contribute to mental disorders like perimenopausal depression.

## Figures and Tables

**Table 1:** Summary of demographic characteristics of the included studies.

Source	Outcomes	Geographic location	No. of sample	Age	Distinguishing menopausal status			Measurement scales
					Pre-	Peri-	Post-	
Punyahotra et al. [40], 1997	Back pain	Thai	248	40-59	127	22	99	Questionnaire
Poomalar and Arounassalame [41], 2013	Muscle and joint pain	Puducherry	500	40-65	NA	135	365	MENQOL
S. A. R. Syed Alwi et al. [42], 2009	Muscles and joints pain	Malaysia	276	47.3–58.2	60	114	102	MENQOL
Blümel et al. [11], 2012	Muscle and joint discomfort	Latin	3373	49.1	1132	1648	1821	MRS
OlaOlorun and Lawoyin [43], 2009	Joint and muscular discomfort	American	1189	40-60	401	68	256	MRS
Sussman et al. [44], 2017	Muscle/joint pain	Nigeria	985	50.85	344	213	497	Questionnaire
Yim et al. [45], 2015	Aching in muscles and joints	Korean	1774	44-56	756	650	368	MENQOL
Ibrahim et al. [29], 2015	Joint and muscular discomfort	Egypt	1214	40-70	503	215	496	MRS
Chou et al. [46], 2014	Joint and muscular discomfort Joint pain	Macau, China	442	40-60	167	124	151	MRS
Islam et al. [47], 2016	Joint and muscular discomfort	USA	1586	30-59	944	133	513	MENQOL
Waidyasekera et al. [48], 2009	Joint and muscular discomfort	USA	683	45-60	144	132	405	MRS
Rahman et al. [49], 2011	Soreness in joints, neck, or shoulder	Bangladesh	509	40-70	122	216	171	MRS
Dugan et al. [14], 2006	Bone and joint discomfort	USA	2218	42-52	307	1642	269	Questionnaire
Khanal [50], 2012	Muscle and joint pain	Nepal	500	45-60	NA	236	264	MRS
Ruan et al. [51], 2016	Muscle and joint discomfort	China	1225	34-76	NA	868	357	Questionnaire
Rathnayake et al., [52], 2019	Muscle and joint pain	Sri Lanka.	350	30-60	184	NA	166	MRS
Thakur et al. [53], 2019	Aches and stiff joints	India	351	35-55	118	117	116	Questionnaire
Szoeke et al. [9], 2008	Muscle and joint discomfort	Australia	438	45-55	NA	NA	NA	Questionnaire
Freeman et al. [15], 2007	Aches, joint pain	USA	404	35-47	368	36	NA	Questionnaire

## Supplementary Materials

Supplementary Materials Detailed descriptions of all database search algorithms, critical appraisal checklist, and the publication bias were included in the Supplementary Materials.

## References

[1] Watt F. E. (2018). Musculoskeletal pain and menopause. *Post Reproductive Health*.

[2] Lubeck D. P. (2003). The costs of musculoskeletal disease: health needs assessment and health economics. *Best Practice & Research Clinical Rheumatology*.

[3] Mäntyselkä P., Kumpusalo E. A., Ahonen R., Takala J. K. (2002). Direct and indirect costs of managing patients with musculoskeletal pain - challenge for health care. *European Journal of Pain*.

[4] Kapur P., Sinha B., Pereira B. M. J. (2009). Measuring climacteric symptoms and age at natural menopause in an Indian population using the Greene Climacteric Scale. *Menopause*.

[5] Epstein S., Sparer E. H., Tran B. N. (2018). Prevalence of work-related musculoskeletal disorders among surgeons and interventionalists: a systematic review and meta-analysis. *JAMA Surgery*.

[6] Soules M. R., Sherman S., Parrott E. (2001). Stages of reproductive aging workshop (STRAW). *Journal of Women's Health & Gender-Based Medicine*.

[7] Ho S. C., Chan S. G., Yip Y. B., Cheng A., Yi Q., Chan C. (1999). Menopausal symptoms and symptom clustering in Chinese women. *Maturitas*.

[8] Delavar M. A., Hajiahmadi M. (2011). Age at menopause and measuring symptoms at midlife in a community in Babol, Iran. *Menopause*.

[9] Szoeke C. E., Cicuttini F. M., Guthrie J. R., Dennerstein L. (2009). The relationship of reports of aches and joint pains to the menopausal transition: a longitudinal study. *Climacteric*.

[10] Gao H. L., Lin S. Q., Wei Y., Chen Y., Wu Z. L. (2013). The effect of age and menopausal status on musculoskeletal symptoms in Chinese women aged 35–64 years. *Climacteric*.

[11] Blumel J. E., Chedraui P., Baron G. (2012). Menopausal symptoms appear before the menopause and persist 5 years beyond: a detailed analysis of a multinational study. *Climacteric*.

[12] Wijnhoven H. A. H., de Vet H. C. W., Picavet H. S. J. (2007). Sex differences in consequences of musculoskeletal pain. *Spine*.

[13] Blümel J. E., Chedraui P., Baron G. (2013). Menopause could be involved in the pathogenesis of muscle and joint aches in mid-aged women. *Maturitas*.

[14] Dugan S. A., Powell L. H., Kravitz H. M., Everson Rose S. A., Karavolos K., Luborsky J. (2006). Musculoskeletal pain and menopausal status. *The Clinical Journal of Pain*.

[15] Freeman E. W., Sammel M. D., Lin H. (2007). Symptoms associated with menopausal transition and reproductive hormones in midlife women. *Obstetrics and Gynecology*.

[16] Frange C., Hachul H., Hirotsu C., Tufik S., Andersen M. L. (2018). Insomnia with musculoskeletal pain in postmenopause: associations with symptoms, mood, and quality of life. *Journal of Menopausal Medicine*.

[17] Egger M., Smith G. D., Schneider M., Minder C. (1997). Bias in meta-analysis detected by a simple, graphical test. *BMJ*.

[18] Harbord R., Harris R., Sterne J., Steichen T. (2000). *METABIAS: Stata module to test for small-study effects in meta-analysis*.

[19] Kontopantelis E., Reeves D. (2009). *METAAN: Stata module to perform fixed- or random-effects meta-analyses*.

[20] DerSimonian R., Laird N. (1986). Meta-analysis in clinical trials. *Controlled Clinical Trials*.

[21] Lewis J. E., Hilditch J. R., Wong C. J. (2005). Further psychometric property development of the Menopause-Specific Quality of Life questionnaire and development of a modified version, MENQOL-Intervention questionnaire. *Maturitas*.

[22] Heinemann L. A. J., Potthoff P., Schneider H. P. (2003). International versions of the menopause rating scale (MRS). *Health and Quality of Life Outcomes*.

[23] Sievert L. L., Goode-Null S. K. (2005). Musculoskeletal pain among women of menopausal age in Puebla, Mexico. *Journal of Cross-Cultural Gerontology*.

[24] Dedicação A. C., de Oliveira Sato T., Avila M. A., Moccellin A. S., Saldanha M. E. S., Driusso P. (2017). Prevalence of musculoskeletal pain in climacteric women of a Basic Health Unit in São Paulo/SP. *Revista Dor*.

[25] Lu J., Liu J., Eden J. (2009). The experience of menopausal symptoms by Arabic women in Sydney. *Climacteric*.

[26] Halbreich U., Kahn L. S. (2007). Atypical depression, somatic depression and anxious depression in women: are they gender-preferred phenotypes?. *Journal of Affective Disorders*.

[27] Olofsson A. S. B., Collins A. (2009). Psychosocial factors, attitude to menopause and symptoms in Swedish perimenopausal women. *Climacteric*.

[28] Mitchell E. S., Woods N. F. (2010). Pain symptoms during the menopausal transition and early postmenopause. *Climacteric*.

[29] Ibrahim Z. M., Sayed Ahmed W. A., El-Hamid S. A., Hagras A. M. (2015). Intimate partner violence among Egyptian pregnant women: incidence, risk factors, and adverse maternal and fetal outcomes. *Clinical and Experimental Obstetrics & Gynecology*.

[30] Andersson G. B. J. (1999). Epidemiological features of chronic low-back pain. *The Lancet*.

[31] Palazzo C., Ravaud J.-F., Papelard A., Ravaud P., Poiraudeau S. (2014). The burden of musculoskeletal conditions. *PloS one*.

[32] Manolagas S. C., O'Brien C. A., Almeida M. (2013). The role of estrogen and androgen receptors in bone health and disease. *Nature Reviews. Endocrinology*.

[33] van Staa T. P., Dennison E. M., Leufkens H. G. M., Cooper C. (2001). Epidemiology of fractures in England and Wales. *Bone*.

[34] Finkelstein J. S., Brockwell S. E., Mehta V. (2008). Bone mineral density changes during the menopause transition in a multiethnic cohort of women. *The Journal of Clinical Endocrinology and Metabolism*.

[35] Lee K. M., Chung C. Y., Kwon S.-S. (2015). Bone mineral density is not associated with musculoskeletal pain in postmenopausal Korean women aged≥50 years. *Clinical Rheumatology*.

[36] Macfarlane T., Blinkhorn A., Worthington H., Davies R. M., Macfarlane G. (2002). Sex hormonal factors and chronic widespread pain: a population study among women. *Rheumatology*.

[37] Santoro N., Brown J. R., Adel T., Skurnick J. H. (1996). Characterization of reproductive hormonal dynamics in the perimenopause. *The Journal of Clinical Endocrinology and Metabolism*.

[38] Shideler S. E., DeVane G. W., Kalra P. S., Benirschke K., Lasley B. L. (1989). Ovarian-pituitary hormone interactions during the perimenopause. *Maturitas*.

[39] Woods N. F., Mitchell E. S. (2005). Symptoms during the perimenopause: prevalence, severity, trajectory, and significance in women's lives. *The American journal of medicine*.

[40] Punyahotra S., Dennerstein L., Lehert P. (1997). Menopausal experiences of Thai women. Part 1: symptoms and their correlates. *Maturitas*.

[41] Poomalar G. K., Arounassalame B. (2013). The quality of life during and after menopause among rural women. *Journal of Clinical and Diagnostic Research*.

[42] Alwi S. A. R. S., Lee P. Y., Awi I., Mallik P. S., Haizal M. N. M. (2009). The menopausal experience among indigenous women of Sarawak, Malaysia. *Climacteric*.

[43] OlaOlorun F. M., Lawoyin T. O. (2009). Experience of menopausal symptoms by women in an urban community in Ibadan, Nigeria. *Menopause*.

[44] Sussman M., Trocio J., Best C. (2015). Prevalence of menopausal symptoms among mid-life women: findings from electronic medical records. *BMC Womens Health*.

[45] Yim G., Ahn Y., Chang Y. (2015). Prevalence and severity of menopause symptoms and associated factors across menopause status in Korean women. *Menopause*.

[46] Chou M. F., Wun Y. T., Pang S. M. (2014). Menopausal symptoms and the menopausal rating scale among midlife Chinese women in Macau, China. *Women & Health*.

[47] Islam R. M., Bell R. J., Billah B., Hossain M. B., Davis S. R. (2016). Prevalence and severity of vasomotor symptoms and joint pain in women at midlife in Bangladesh: a population-based survey. *Menopause*.

[48] Waidyasekera H., Wijewardena K., Lindmark G., Naessen T. (2009). Menopausal symptoms and quality of life during the menopausal transition in Sri Lankan women. *Menopause*.

[49] Rahman S., Salehin F., Iqbal A. (2011). Menopausal symptoms assessment among middle age women in Kushtia, Bangladesh. *BMC Research Notes*.

[50] Khanal M. R. (2012). Study of menopausal symptoms among peri and postmenopausal women attending NMCTH. *Nepal Medical College journal*.

[51] Ruan X., Cui Y., du J., Jin F., Mueck A. O. (2017). Prevalence of climacteric symptoms comparing perimenopausal and postmenopausal Chinese women. *Journal of Psychosomatic Obstetrics & Gynecology*.

[52] Rathnayake N., Lenora J., Alwis G., Lekamwasam S. (2019). Prevalence and severity of menopausal symptoms and the quality of life in Middle-aged Women: a study from Sri Lanka. *Nursing Research and Practice*.

[53] Thakur M., Kaur M., Sinha A. (2019). Assessment of menopausal symptoms in different transition phases using Greene Climacteric Scale among rural women of North India. *Annals of Human Biology*.

