# Supplementary material for: Musculoskeletal Pain during the Menopausal Transition: A Systematic Review and Meta-Analysis
Source: Neural Plast. 2020 Nov 25;2020:8842110. doi: 10.1155/2020/8842110 (PMC7710408; doi:10.1155/2020/8842110)
Supplement: Supplementary Materials — Detailed descriptions of all database search algorithms, critical appraisal checklist, and the publication bias were included in the Supplementary Materials. [file 8842110.f1.docx]

**Supplementary Materials:**

**Database Search Algorithms**

Medline:

exp Menopause/ or exp Climacteric/ or *Female/ or exp Postmenopause/ or exp Premenopause/ or exp perimenopause or (Menopause or Climacteric or Postmenopause or Premenopause or Perimenopause. ti,ab) AND (Musculoskeletal Pain/ or *Musculoskeletal Diseases/ or *Female/ or *Pain/ or (muscle or joint pain).ti,ab or (muscle or joint discomfort).ti,ab.)

Web of Science

TS=("menopausal women" OR "climacteric women " OR "women, transition period" OR "postmenopause" OR "premenopause" OR "perimenopause" OR "menopausal transition" ) AND TS=("musculoskeletal Diseases" OR "musculoskeletal symptom" OR "musculoskeletal disorder" OR "musculoskeletal symptom" OR "musculoskeletal pain" OR "musculoskeletal complaint" OR "muscle or joint pain" OR "muscle or joint discomfort" )

Pubmed

((Musculoskeletal symptom*[tiab] OR musculoskeletal disorder*[tiab] OR musculoskeletal pain [tiab] OR musculoskeletal complaint*[tiab] OR "muscle/joint pain" [tiab] OR "muscle/joint discomfort" [tiab])) AND (("menopausal women"[mesh] OR "climacteric women "[mesh] OR “women, transition period” [mesh] OR "Premenopause"[Mesh] OR "Perimenopause"[Mesh] OR "postmenopause"[Mesh] OR "premenopause"[tiab] OR "Perimenopause"[tiab] OR "postmenopause"[tiab] OR "menopausal women"[tiab] OR "climacteric women "[tiab] OR “women, transition period” [tiab] OR “menopausal transition” [tiab]))

Embase

(('menopausal female'/exp/mj OR 'menopausal female':ab,ti OR 'climacteric female'/exp/mj OR 'climacteric female':ab,ti OR 'premenopausal' OR 'premenopausal female':ab,ti) AND 'perimenopausal female'/exp/mj OR 'perimenopausal female':ab,ti OR 'postmenopausal female'/exp/mj OR 'postmenopausal female':ab,ti) AND ('musculoskeletal diseases'/exp/mj OR 'musculoskeletal diseases':ab,ti OR 'musculoskeletal symptom'/exp/mj OR 'musculoskeletal symptom':ab,ti OR 'musculoskeletal disorders'/exp/mj OR 'musculoskeletal disorders':ab,ti OR 'musculoskeletal pain'/exp/mj OR 'musculoskeletal pain':ab,ti OR ('muscle'/exp/mj AND 'joint pain'/exp/mj) OR (muscle:ab,ti AND 'joint pain':ab,ti)) AND [embase]/lim AND [female]/lim

**Table S1:**. Critical appraisal checklist from the Joanna Briggs Institute (JBI) Reviewers’ Manual 2014 for the systematic review of prevalence and incidence Data.

| Were  objective,  standard  criteria used for the  measurement of the  condition? | yes | yes | yes | yes | Yes | yes | yes |
| --- | --- | --- | --- | --- | --- | --- | --- |
| Was the  data  analysis  conducted with sufficient coverage of the  identified sample？ | Unclear | Yes | Unclear | Unclear | Yes | Unclear | Yes |
| Were the  study  subjects  and setting described in detail？ | Yes | Yes | Yes | Yes | Yes | Unclear | Unclear |
| Were study  subjects  recruited in  an  appropriate  way? | Yes | Unclear | Unclear | Yes | Yes | Unclear | Yes |
| Was the  Sample size  adequate？ | Yes | Yes | Yes | Yes | Yes | Yes | Yes |
| Was the  sample  representative of the  target  population？ | Unclear | Unclear | NA | Yes | Yes | Yes | Yes |
| Was there  appropriate  statistical  analysis? | Yes | Yes | Yes | Yes | Yes | Yes | Yes |
| Are all important  Confounding factors,  subgroups, and  differences  identified and  accounted for? | Unclear | Unclear | Yes | Unclear | Unclear | Yes | NA |
| Were  subpopulations  identified using  objective  criteria? | Yes | Yes | Yes | Yes | Yes | Yes | Yes |
| Study | Poomalar et al. 2013 | Blümel et al., 2012 | Szoeke et al.,2008 | Lan et al., 2017 | Islam et al., 2016 | Yim et al., 2015 | Funmilola et al., 2009 |

| yes | Yes | yes | yes | yes | unclear | Yes | yes | Yes | yes | Yes | yes |
| --- | --- | --- | --- | --- | --- | --- | --- | --- | --- | --- | --- |
| Unclear | Yes | Yes | Yes | Yes | Yes | Yes | Yes | Yes | Yes | Yes | Yes |
| Unclear | Unclear | Unclear | Unclear | Unclear | Unclear | Yes | Unclear | Unclear | Unclear | Unclear | Unclear |
| Yes | Yes | Yes | Yes | Yes | Unclear | Yes | Yes | Yes | Yes | Yes | Yes |
| Yes | Yes | Yes | Yes | Yes | Yes | Yes | Yes | Yes | Yes | Yes | Yes |
| Yes | Yes | Yes | Yes | Yes | Yes | Yes | Yes | Yes | Yes | Yes | Yes |
| Yes | Yes | Yes | Yes | Yes | Yes | Yes | Yes | Yes | Yes | Yes | Yes |
| NA | Yes | NA | Yes | NA | NA | Yes | NA | Yes | NA | Yes | NA |
| NA | Yes | Yes | Yes | Yes | Yes | Yes | Yes | Yes | Yes | Yes | Yes |
| Rathnayake et al.,2019 | Ibrahim et al., 2015 | Chou et al., 2014 | Waidyasekera et al., 2009 | Rahman et al., 2011 | Punyahotra et al.1997 | Dugan et al., 2006 | Freeman et al,2007 | Thakur et al.,2019 | Ruan et al.,2016 | S. A. R. Syed, 2009 Alwi.[44], 2009 | Khanal et al, 2012 |


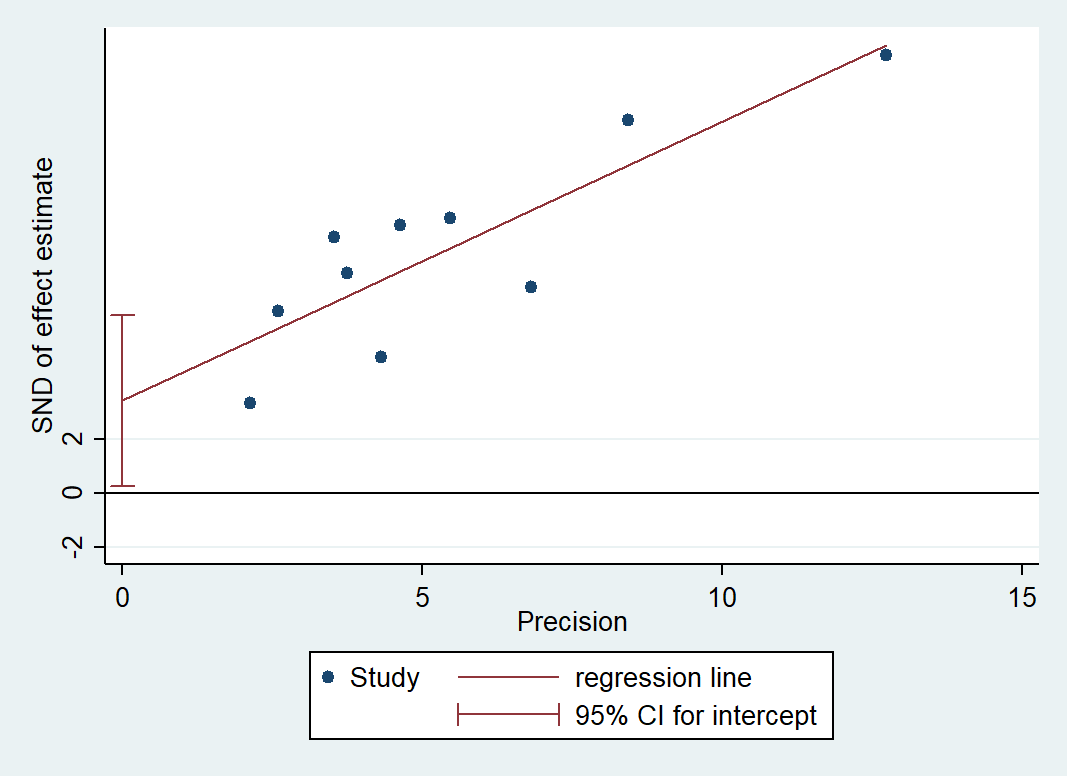


**Figure S1.** The publication bias in the pre- vs peri-menopausal status analysis **(**intercept of the regression a = 2.5, t = 4.67, *P* = 0.037; SND, standard normal deviate**).** The publication bias was significant for the studies included in the pre- vs perimenopausal state analysis.


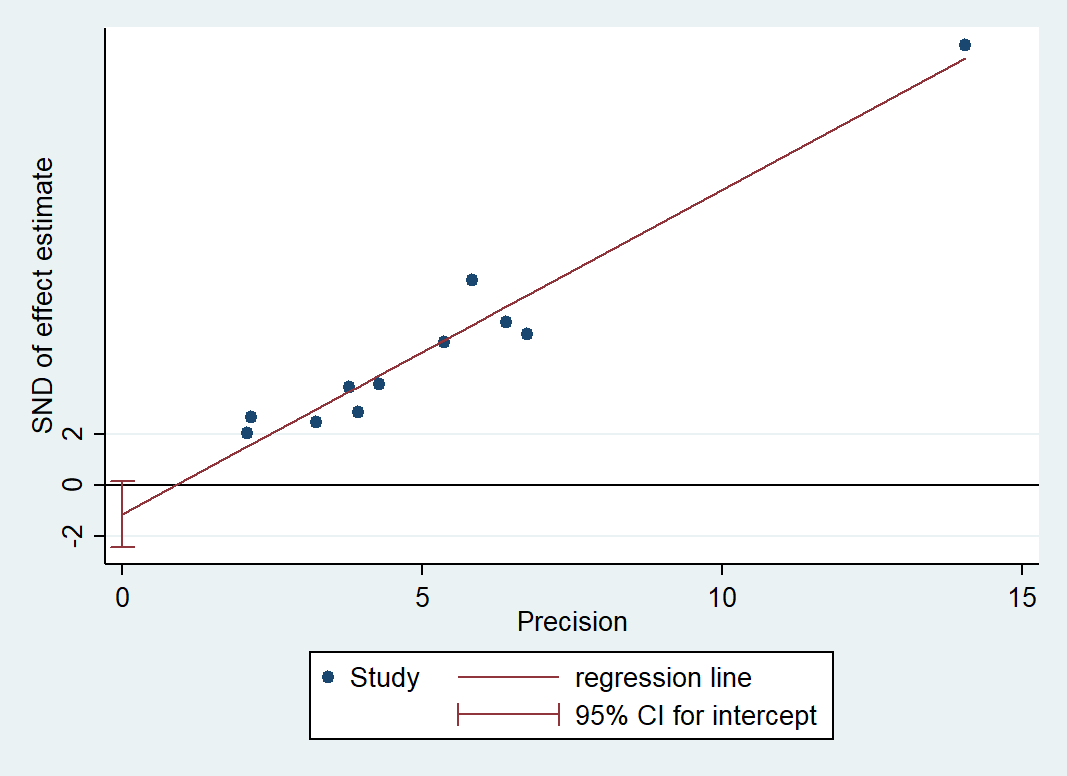


**Figure S2.** The publication bias in the peri- vs postmenopausal state analysis **(**intercept of the regression a = -1.98, t = 13.54, *P* = 0.079; SND, standard normal deviate**).** The publication bias was not significant for the studies included in the peri- vs postmenopausal state analysis.
